# Different Responses of Soil Bacterial and Fungal Communities to 3 Years of Biochar Amendment in an Alkaline Soybean Soil

**DOI:** 10.3389/fmicb.2021.630418

**Published:** 2021-05-26

**Authors:** Wenhui Gao, Ke Gao, Zonghao Guo, Yuan Liu, Li Jiang, Cheng Liu, Xiaoyu Liu, Guangli Wang

**Affiliations:** ^1^Department of Bioengineering, College of Life Science, Huaibei Normal University, Huaibei, China; ^2^State Key Laboratory of Desert and Oasis Ecology, Xinjiang Institute of Ecology and Geography, Chinese Academy of Sciences, Urumqi, China; ^3^Institute of Resources, Ecosystem and Environment of Agriculture, Nanjing Agricultural University, Nanjing, China

**Keywords:** soil fungi, rhizosphere, soybean field, microbial community, biochar

## Abstract

Biochar as a soil amendment has been regarded as a promising way to improve soil fertility. However, the response of microbial community after biochar and biochar compound fertilizer (BCF) application has not been thoroughly elucidated. This study evaluated the changes in abundance and composition of bacterial and fungal communities using quantitative real-time PCR (qPCR) and Illumina MiSeq amplicon sequencing. The field experiment ran for 3 years and comprised five treatments: chemical fertilizer as control (CK), straw-returning combined with chemical fertilizer (CS), low biochar application combined with chemical fertilizer (LB), high biochar application combined with chemical fertilizer (HB) and BCF. The results showed that biochar amendment results no changes in the abundance and diversity of bacteria in the bulk and rhizosphere soils. However, the abundance of soil fungi was significantly increased by biochar amendment (LB and HB). LB treatment significantly increased the fungal alpha diversity, while there was no significant change under HB. Furthermore, the dominant bacterial phyla found in the samples were *Proteobacteria*, *Actinobacteria*, and *Acidobacteria*. Biochar addition increased the relative abundance of *Actinobacteria* in both bulk and rhizosphere soils. The dominant fungal phyla were *Ascomycota*, *Mortierellomycota*, and *Basidiomycota*. The relative abundance of *Ascomycota* significantly decreased, but *Mortierellomycota* significantly increased in LB and HB. In addition, redundancy analysis indicated that the changes in bacterial and fungal communities are associated with soil properties such as SOC and TN, which are crucial contributors in regulating the community composition. This study is expected to provide significant theoretical and practical knowledge for the application of biochar in agricultural ecosystem.

## Introduction

Biochar is a type of solid product obtained by the thermal decomposition of biomass such as manure, crop straws and sewage sludge under aerobic or anoxic conditions (Lehmann et al., 2006). As an emerging soil improving material, biochar has some excellent physicochemical properties, like high pH, high carbon content, high surface area, high porosity and large cation adsorption ability, varying depending upon the raw materials and pyrolysis processes (Sun et al., 2016; Zheng et al., 2016; Yao et al., 2017b). Recently, the application of biochar amendment to soil has received increasing attention due to its positive influence on improving soil carbon storage and fertility (Yao et al., 2017a; Zhang et al., 2017; Cheng et al., 2019). In previous studies, Robertson et al. (2012) and Tong et al. (2014) reported that biochar positively promoted plant growth and increased crop yields. In addition, the emission of N_2_O from soil significantly decreased after biochar addition, as observed in many studies (Ameloot et al., 2013; Liu et al., 2014; Agegnehu et al., 2016; Krause et al., 2018). However, when compared with the studies on the effects of biochar on the soil physicochemical properties, the effects on soil microbial community in long-term cropland have received much less attention (Zheng et al., 2016; Yu et al., 2018).

Soil microorganisms play a pivotal role in processes such as nutrient cycling and mineralization (Zheng et al., 2016). Changes in microbial community structure, owing to sensitivity to environmental conditions, might indicate the potential effects of biochar (Liu et al., 2019). Soil bacteria and fungi play an important role in soil nutrient transformation and can decompose recalcitrant matter such as cellulose and lignin (Gul et al., 2015; Yao et al., 2017a). The rhizosphere is an area of soil around plant roots (Egamberdieva et al., 2008; Mendes et al., 2011). Generally, microbial communities in the rhizosphere are affected by the root exudates and plant residues (Berendsen et al., 2012; Yu et al., 2018). Thus, the activities of microbes and enzymes in the rhizosphere are much higher than those in the bulk soil. In a short-term field trial, Yu et al. (2018) revealed that total microbial biomass increased after N-biochar addition, while the ratio of G+/G− bacterial Phospholipid fatty acids (PLFAs) in the rhizosphere was lower than in bulk soil. The majority of previous studies on biochar were carried out in a greenhouse using laboratory incubation in pots or in short-term field trials (Yao et al., 2017a; Zhou et al., 2020).

Elzobair et al. (2016) suggested that biochar application (22 t⋅ha^–1^) had no significant effect on microbial community structure and extracellular enzyme activities when compared with manure amendment during a short-term experiment. This could be attributed to the relatively low application rate of biochar. However, Chen et al. (2018) showed that the addition of biochar (10–15%) altered microbial community structure and significantly increased the richness and diversity index of total microbes. Zhou et al. (2020) found that the addition of leaf and woodchip biochar increased the abundance of P-solubilizing bacteria and diversity of soil bacterial community in the forest soil. These inconsistent results could account for the discrepancy in plant species, biochar characteristics, soil type and biochar application period (Sun et al., 2016; Zheng et al., 2016; Yao et al., 2017b; Cheng et al., 2019).

When considering the physical form of biochar, the powdered form is difficult to apply, transport and store. Moreover, the nutrient content of powdered biochar is insufficient and the amount reaching the crops is limited. Biochar compound fertilizer (BCF) was produced by mixing biochar with chemical fertilizer and then fixing into small granules. Therefore, the BCF is expected to the options of agricultural fertilization. Zheng et al. (2017b) found that BCF significantly increased maize yield by 10.7% and carbon efficiency by 46.2% in a field experiment. Compared to corn straw and pig manure compost treatments, biochar-based compound fertilizer did not significantly improve soil structure but improved the accumulation of SOC (Chen et al., 2020). However, there are relatively few studies on the effect of BCF application on soil microbial community. Biochar-fertilizer interaction significantly increased the abundance and altered community structure of bacteria in the rhizosphere when compared with the no fertilizer control (Ibrahim et al., 2020).

Here, we conducted a field study to elucidate the effects of crop straw, biochar and BCF on soil properties and microbial communities. We hypothesized that biochar and its induced changes on soil physicochemical properties and microbial community structure can be retained in the soil for several years. In this study, we used quantitative real-time PCR (qPCR) and Illumina MiSeq amplicon sequencing to evaluate the abundances, community composition and structure of soil bacteria and fungi in the bulk and rhizosphere soil following the application of different biochar applications.

## Materials and Methods

### Site Description and Experiment Design

The experimental site is located in Xuji village (34°04′N, 116°93′E) in Huaibei Municipality of Anhui Province, China, lying in Huang-Huai-Hai Plain. The experiment was conducted in June of 2017. In this area, a wheat-soybean rotation was being implemented as the traditional cropping system. During the last 10 years, the area has been witnessing four distinct seasons and a typical temperate monsoon climate. The annual average temperature of the region was 14.8°C, the annual mean precipitation was 830 mm, and the annual frost-free period was 202 days. Field management was carried out by following local cultivars and conventional crop practices, including fertilization, insecticide application and weed control. The soil texture was sandy loam, derived from fluvio lacustrine sediments and classified as Vertisol. The soil had a pH (H_2_O) of 8.05, soil organic carbon (SOC) of 11.35 g⋅kg^–1^, total N (TN) of 0.98 g⋅kg^–1^, available P (AP) and K (AK) of 14.61 and 120.29 mg⋅kg^–1^, respectively.

The experiment was composed of five treatments: control (CK) with chemical fertilizer, wheat crop straw addition plus chemical fertilizer (CS), low biochar addition at 7 t⋅ha^–1^ plus chemical fertilizer (LB), high biochar addition at 20 t⋅ha^–1^ plus chemical fertilizer (HB) and BCF. Each treatment was replicated three times on a single plot of 20 m^2^ (4 m × 5 m) in area, set out in a randomized block design. The biochar was evenly spread on the soil surface and tilled into the soil of approximately 20 cm in June 2017. For the HB treatment, biochar was applied once at 20 t⋅ha^–1^, while the biochar in LB treatment was added at 3 t⋅ha^–1^ for soybean and 4 t⋅ha^–1^ for wheat season. The crop straw was chopped into small pieces and then spread on the soil surface. Biochar used in this experiment was purchased from Nanjing Qinfeng Crop Straw Technology Company, China. The rice straw was firstly chopped into small pieces and then pyrolyzed in a rotational kiln at 550–650°C under oxygen-deficient conditions. After cooling, biochar samples were ground into powder, sieved (< 2 mm) and measured their physiochemical properties. The biochar has a pH (H_2_O) of 10.07, organic C of 470.70 g⋅kg^–1^, total N of 9.19 g⋅kg^–1^, total P of 6.01 g kg^–1^, total K 19.12 g kg^–1^, ash of 37.4%, electric conductivity of 139.75 μs cm^–1^. The BCF contained 15% of N, 15% of P_2_O_5_, 10% of K_2_O and 40% of C contents. In the experiment, all treatments had the same amounts of total N, P and K applied. For the treatments with chemical fertilizer (CK, CS, LB, and HB), N fertilizer was applied at 112.5 kg N ha^–1^ as a basal fertilizer, P and K fertilizers were applied at 112.5 kg P_2_O_5_ ha^–1^ and 112.5 kg K_2_O ha^–1^, respectively. For the BCF treatment, BCF was applied at 750 kg⋅ha^–1^. The management practices of applying pesticide and herbicide were consistent across all treatments.

### Soil Sampling and Soil Properties Analyses

The soil samples were collected 1 week before soybean harvest in October of 2019. For the rhizosphere soil, 6–8 individual crops were randomly selected from each plot. After gentle shaking off the loosely adhered root soil, the remaining soil (about 1–2 cm thickness) was homogenized to form one composite sample. For the bulk soil, ten cores (5 cm in diameter) adjacent to the crops were randomly collected from the plow layer soil (0–15 cm) in each plot with a drill, and then mixed uniformly to form one composite sample. The 30 composite soil samples, including 15 samples of bulk and rhizosphere, respectively, were transferred to sterile plastic bags, sealed and placed on ice to be transported to the laboratory. The soil samples were sieved through 2-mm mesh and plant residues and gravels were removed. A part of the soil samples was air-dried at room temperature to soil physicochemical analyses, the other part sample was stored at −20°C to DNA extraction within 1 week.

Soil pH was determined at a soil-to-water ratio of 1:2.5 using a pH meter (Mettler Toledo FE20, China). Soil NH_4_^+^ and NO_3_^–^ were extracted with 2 M KCl and their concentrations in the extracts were determined at 25°C by a Continuous Flow Chemical Analytical System (TRAACA-2000, Germany). SOC and TN were determined by wet digestion with K_2_Cr_2_O_7_ oxidation and semi-Kjeldahl method, respectively. AP and AK were measured by colorimetric assay and flame photometer method, respectively.

### Soil DNA Extraction and Quantitative PCR

Total DNA was extracted from 0.5 g bulk and rhizosphere soil using a PowerSoil^TM^ DNA Isolation Kit (MoBio Laboratories Inc., CA, United States), according to the manufacturer’s instructions. The DNA was purified through SanPrep Column PCR Product Purification Kit (Sangon Biotech Co., China). The concentration and quality of the extracted DNA were assessed using a NanoDrop ND-1000 Spectrophotometer (NanoDrop Technologies, United States).

The qPCR was performed in a Roche LightCycler 480 system (Roche Diagnostics, Germany) via fluorometric monitoring with SYBR Green 1 dye. The primer pairs 338F/518R (Fierer et al., 2005) and 5.8s/ITS1F (Yao et al., 2017b) were used to quantify bacterial 16S rRNA and fungal ITS genes in all samples, respectively. Each reaction was performed in a 20 μL volume mixture containing 10 μL of 2 × Universal SYBR Green Fast qPCR Mix (ABclonal Technology, United States), 0.5 μL each of 10 μM forward and reverse primers, 1.0 μL of standard or extracted soil DNA, and 8.0 μL sterilized water. The plasmid DNA preparation was obtained from the clone with the correct insert using a Miniprep kit (Qiagen, Germantown, United States). The plasmid concentrations were measured using a NanoDrop ND-1000 Spectrophotometer (NanoDrop Technologies, United States) and then 10-fold serially diluted for the standard curves. Melting curve analysis of qPCR products was conducted following each assay to confirm that specific amplification was not from primer-dimers or other impurities. Amplification efficiencies of 95–104% were obtained with R^2^-values over 0.98.

### Illumina MiSeq Amplicon Sequencing and Data Analysis

The community compositions of both bacteria and fungi in each plot were identified by the Centre for Genetic and Genomic Analysis, GENESKY Biotechnologies Inc. (Shanghai, China). The DNA of each soil sample was used as a template for PCR amplification. Moreover, the bacterial 16S rRNA V4-V5 and fungal ITS1 were amplified using primer pairs 515F/907R and ITSI/ITS2, respectively. Each sample was amplified in triplicates. At the same time, standard genomic DNA of the bacteria and fungi was used as a positive control. The PCR products were checked in a 1.5% agarose gel electrophoresis to assess their specificity. These were then purified using Agencourt AMpure XP PCR Purification Beads (Sangon Biotech Co., China). Purified amplicons were pooled in equimolar and paired-end sequenced (2 × 250 bp) using Illumina Miseq amplicon sequencing.

The raw sequencing data were processed and trimmed using Quantitative Insights Into Microbial Ecology (QIIME) software to remove the low-quality sequences (quality score < 20), primers, barcodes, adaptors (Caporaso et al., 2010), and chimeras were detected and removed using the UCHIME algorithm (Edgar et al., 2011). The remaining high-quality sequences were clustered into Operational Taxonomic Units (OTUs), with a 97% similarity cutoff. The representative sequences for each OTU were aligned using the Python Nearest Alignment Space Termination (PyNAST) (DeSantis et al., 2006; Yao et al., 2017a). The taxonomy of each depty phylotype was assigned using a BLAST comparison against sequences within the GenBank database. Rarefaction curves and alpha diversity indices were calculated using QIIME software based on the obtained OTUs. All sequences have been deposited in the National Center for Biotechnology Information (NCBI) Short Reads Archive database SRP307320.

### Statistical Analysis

Statistical analysis was performed using the SPSS Version 20.0 for Windows. The data are checked for normality and homogeneity of variance (Duncan’s test) before analysis of variance (ANOVA) and were subsequently transformed to meet the assumptions of the ANOVA analyses if necessary. Significant differences in soil properties, gene copy number, alpha diversity and relative abundances among the treatments in the bulk and rhizosphere were identified separately by One-way ANOVA followed by Duncan’s-test (*p* < 0.05). Pearson’s correlation was used to determine the relationships between gene copy number and soil properties. OTUs were analyzed for alpha and beta diversity for bacteria and fungi. Non-metric multidimensional scaling (NMDS) plots based on Bray-Curtis distance were generated to represent the differences in microbial community between different treatments. Redundancy analysis (RDA) was used to assay the relationships between microbial community and soil properties. The NMDS and RDA were performed using the “vegan” package in R (R Development Core Team, 2015).

## Results

### Soil Physicochemical Properties

Soil physicochemical properties of the treatments are shown in Table 1. The soil was alkaline with pH ranging from 8.15 to 8.20 in the bulk soil, while between 8.13 and 8.18 in the rhizosphere. In general, biochar application induces significant changes in most soil parameters (*p* < 0.05) except in soil pH, NH_4_^+^ and NO_3_^–^. The amendment of biochar or BCF greatly increased the content of SOC and AP in the bulk soil as compared to CK. The concentration of TN was significantly increased with HB treatment, while there was no significant difference with other treatments compared to CK. The level of AK was higher in HB than the control, while no remarkable changes were observed with LB and BCF when compared to CK. A similar trend was observed in the rhizosphere.

**TABLE 1:** Soil physiochemical properties in the bulk and rhizosphere soils of soybean.

	Treatments	pH	SOC (g⋅kg^–1^)	TN (g⋅kg^–1^)	NH_4_^+^ (mg⋅kg^–1^)	NO_3_^–^ (mg⋅kg^–1^)	AP (mg⋅kg^–1^)	AK (mg⋅kg^–1^)
Bulk	CK	8.19 ± 0.02a	9.95 ± 2.04c	0.98 ± 0.02b	3.96 ± 0.47ab	1.85 ± 0.53a	13.92 ± 0.79b	159.87 ± 16.10c
	CS	8.17 ± 0.04a	12.36 ± 1.71b	0.99 ± 0.03b	3.99 ± 0.26ab	1.95 ± 0.39a	16.19 ± 0.78a	199.47 ± 19.17b
	LB	8.17 ± 0.03a	12.85 ± 1.56b	1.01 ± 0.01b	4.07 ± 0.59ab	1.76 ± 0.45a	16.21 ± 0.64a	167.83 ± 9.62bc
	HB	8.20 ± 0.02a	15.25 ± 1.42a	1.07 ± 0.02a	3.43 ± 0.31b	1.75 ± 0.58a	16.58 ± 0.68a	279.63 ± 14.63a
	BCF	8.15 ± 0.03a	12.75 ± 0.67b	1.00 ± 0.05b	4.45 ± 0.47a	2.06 ± 0.37a	17.05 ± 0.55a	154.73 ± 17.45c
Rhizosphere	CK	8.16 ± 0.03A	10.04 ± 2.08C	1.02 ± 0.01B	3.87 ± 0.37AB	1.95 ± 0.63A	14.12 ± 0.89B	156.77 ± 15.10C
	CS	8.15 ± 0.05A	12.49 ± 1.81B	1.04 ± 0.03B	3.84 ± 0.20AB	2.04 ± 0.79A	16.69 ± 0.88A	195.87 ± 16.17B
	LB	8.17 ± 0.02A	12.93 ± 1.26B	1.03 ± 0.02B	3.97 ± 0.69AB	1.86 ± 0.65A	16.71 ± 0.74A	165.63 ± 9.62BC
	HB	8.18 ± 0.02A	15.33 ± 1.92A	1.12 ± 0.02A	3.26 ± 0.31B	1.89 ± 0.55A	16.68 ± 0.78A	272.13 ± 16.63A
	BCF	8.13 ± 0.01A	12.82 ± 0.67B	1.02 ± 0.07B	4.34 ± 0.67A	2.21 ± 0.47A	17.23 ± 0.75A	149.23 ± 19.45C

*Different letters within the same column indicate significant differences between the treatments (p < 0.05), lowercase letters for the bulk soil and uppercase letters for the rhizosphere. CK, control; CS, wheat straw addition; LB, low biochar addition; HB, high biochar addition; BCF, biochar compound fertilizer. SOC, soil organic carbon; TN, total nitrogen; AP, available phosphorus; AK, available potassium.*

### Abundances of Soil Bacteria and Fungi

**FIGURE 1:**
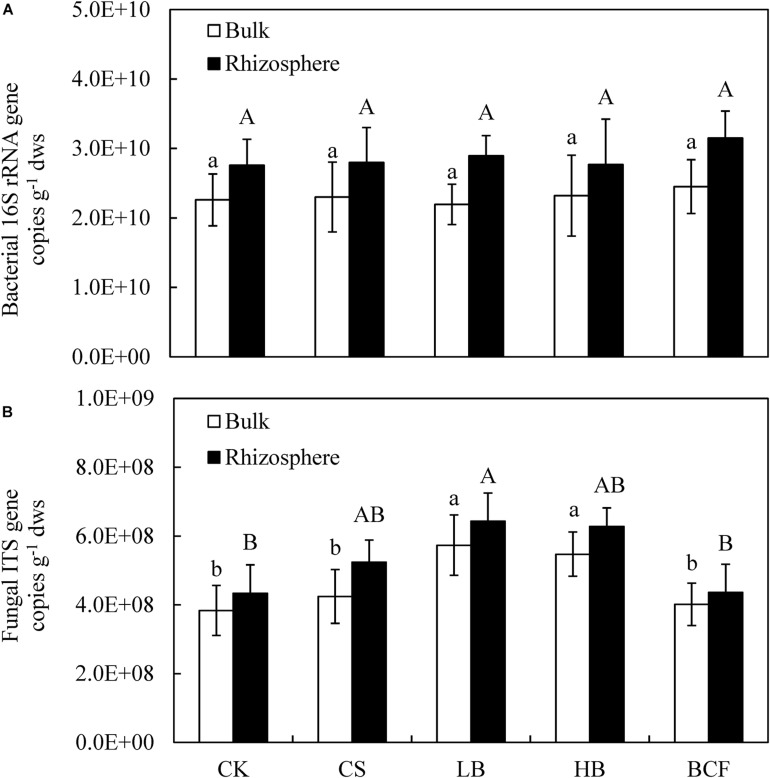
The abundance of bacterial 16S rRNA **(A)** and fungal ITS1 **(B)** gene copies in the bulk and rhizosphere soils of soybean. Different letters above the columns denote significant differences among the treatments in the bulk or rhizosphere soil at *p* < 0.05, lowercase letters for the bulk soil and uppercase letters for the rhizosphere. CK, control; CS, wheat straw addition; LB, low biochar addition; HB, high biochar addition; BCF, biochar compound fertilizer.

The abundance of soil bacterial 16S rRNA genes in all treatments ranged from 2.19 × 10^10^ to 3.15 × 10^10^ copies g^–1^ dry soil, were 19.40–31.96% higher in the rhizosphere than in bulk soil (Figure 1A). With the amendment of biochar or BCF the abundance was slightly higher than in the CK treatment, while the difference was not statistically significant (*p* > 0.05) regardless of the bulk or rhizosphere soil. The copy number of fungal ITS genes ranged from 3.84 × 10^8^ to 6.43 × 10^8^ copies g^–1^ dry soil, were 8.71–23.56% higher in the rhizosphere than in bulk soil (Figure 1B). In the bulk soil, the amendment of biochar (LB and HB) significantly increased the fungal abundance compared to CK. In addition, LB also greatly increased rhizosphere fungal abundance. Moreover, the Pearson’s correlation analysis showed that fungal gene abundance a significant positive correlated with SOC (*p* < 0.01) in bulk and rhizosphere soil, while the same correlation was observed with TN (*p* < 0.05) in the bulk soil (Table 2).

**TABLE 2:** Pearson’s correlation coefficients between soil characteristics, bacterial, and fungal gene abundances.

		pH	SOC	TN	NH_4_^+^	NO_3_^–^	AP	AK
Bulk	Bacterial gene abundance	–0.022	0.142	0.067	–0.275	–0.065	0.192	0.114
	Fungal gene abundance	0.383	0.708**	0.624*	–0.203	0.252	0.007	–0.513
Rhizosphere	Bacterial gene abundance	–0.112	0.011	0.046	0.427	0.286	0.154	–0.136
	Fungal gene abundance	0.431	0.652**	0.257	–0.304	–0.240	0.220	0.469

* and ** represent the significant correlation at p < 0.05 and p < 0.01, respectively.

### Diversity and Composition of Bacterial and Fungal Communities

**FIGURE 2:**
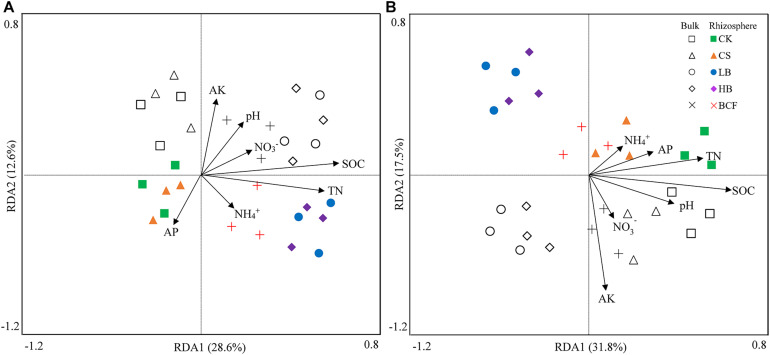
Relative abundance (%) of the dominant phyla of bacteria **(A)** and fungi **(B)** in the bulk and rhizosphere soils of soybean. Different letters above the columns denote significant differences among the treatments in the bulk or rhizosphere soil at *p* < 0.05, lowercase letters for the bulk soil and uppercase letters for the rhizosphere. CK, control; CS, wheat straw addition; LB, low biochar addition; HB, high biochar addition; BCF, biochar compound fertilizer.

After sequence quality control, a total of 3,298,552 bacterial sequences and 4,656,942 fungal sequences were obtained from the 30 soil samples. The number of OTUs ranged from 4,428 to 4,764 of bacteria and 479–585 OTUs of fungi at a 97% similarity (Table 3). When compared to CK, straw and biochar treatments had no significant effects on the alpha diversity, including Chao1, ACE and Shannon (*p* > 0.05). The dominant bacterial phyla (relative abundance > 5%) across all soil samples were *Proteobacteria*, *Actinobacteria*, *Acidobacteria*, *Planctomycetes*, *Chloroflexi* and *Bacteroidetes*, with relative abundances ranging from 26.80 to 33.95%, 11.25 to 21.43%, 9.01 to 14.58%, 8.55 to 11.56%, 6.66 to 8.95% and 4.29 to 7.63%, respectively (Figure 2A). In addition, the phyla of *Firmicutes*, *Thaumarchaeota*, *Gemmatimonadetes*, and *Nitrospirae* were less abundant (relative abundance > 0.1% but < 5%), but were still detected across all samples (data not shown). The application of biochar (LB and HB) increased the relative abundance of *Actinobacteria* in both bulk and rhizosphere when compared with CS. And LB treatment significantly decreased the relative abundance of *Bacteroidetes* compared in the bulk soil, while other main phyla did not show significant change with any treatment used. Further taxonomical classification at the family level revealed that 11 family (with relative abundance > 1%) were detected in all soil samples. Among them, the family *Planctomycetaceae*, *Rhizobiaceae*, *Nitrososphaeraceae*, *Nocardioidaceae*, *Gaiellaceae*, *Anaerolineaceae*, and *Chitinophagaceae* were abundant (relative abundance > 2%) in the soybean bulk and rhizosphere soil (Supplementary Figure 2). LB significantly increased the relative abundance of *Rhizobiaceae* but decreased the relative abundance of *Chitinophagaceae* in the rhizosphere.

**TABLE 3:** Richness and diversity indices of soil bacterial and fungal communities in the bulk and rhizosphere soils of soybean.

	Treatments	Bacteria					Fungi				
		Clean reads	OTUs	Chao1	ACE	Shannon	Clean reads	OTUs	Chao1	ACE	Shannon
Bulk	CK	112,486 ± 4,206	4,744 ± 140a	7,053 ± 152a	7,050 ± 133a	7.01 ± 0.08a	175,749 ± 52,675	508 ± 21b	1,040 ± 72b	1,048 ± 78b	3.60 ± 0.22b
	CS	113,789 ± 16,242	4,731 ± 110a	6,982 ± 154a	6,930 ± 117a	7.04 ± 0.11a	139,701 ± 4,314	543 ± 22ab	1,048 ± 24b	1,056 ± 32b	3.79 ± 0.16ab
	LB	110,023 ± 8,862	4,737 ± 74a	6,849 ± 69a	6,855 ± 93a	7.03 ± 0.05a	197,950 ± 55,617	585 ± 26a	1,298 ± 21a	1,303 ± 41a	4.16 ± 0.12a
	HB	107,440 ± 1,395	4,753 ± 106a	6,836 ± 171a	6,842 ± 157a	7.06 ± 0.06a	173,713 ± 34,402	575 ± 20ab	1,170 ± 39ab	1,163 ± 63ab	4.09 ± 0.12a
	BCF	89,193 ± 14,051	4,764 ± 43a	6,981 ± 52a	6,968 ± 69a	7.05 ± 0.02a	165,003 ± 39,064	526 ± 36ab	1,070 ± 80b	1,068 ± 86b	3.87 ± 0.19ab
Rhizosphere	CK	104,753 ± 5,742	4,547 ± 249A	6,736 ± 378A	6,756 ± 370A	6.96 ± 0.28A	155,245 ± 31,684	479 ± 25A	960 ± 65B	929 ± 44B	3.50 ± 0.15B
	CS	96,482 ± 11,357	4,606 ± 68A	6,798 ± 49A	67,829 ± 58A	7.06 ± 0.06A	115,689 ± 10,245	495 ± 25A	1,029 ± 89AB	1,039 ± 90AB	3.46 ± 0.22B
	LB	105,437 ± 8,537	4,428 ± 57A	6,589 ± 213A	6,552 ± 161A	6.83 ± 0.15A	168,532 ± 36,789	512 ± 22A	1,192 ± 94A	1,171 ± 106A	3.94 ± 0.09A
	HB	97743 ± 4385	4,515 ± 176A	6,604 ± 264A	6,587 ± 238A	7.01 ± 0.12A	147,589 ± 23,467	492 ± 35A	1,072 ± 48AB	1,055 ± 70AB	3.75 ± 0.08AB
	BCF	82,253 ± 18,051	4,577 ± 193A	6,785 ± 62A	6,739 ± 43A	7.06 ± 0.02A	149,234 ± 30,368	479 ± 38A	1,007 ± 92AB	1,008 ± 99AB	3.39 ± 0.15B

*Different letters within the same column indicate significant differences among the treatments at p < 0.05, lowercase letters for the bulk soil and uppercase letters for the rhizosphere. CK, control; CS, wheat straw addition; LB, low biochar addition; HB, high biochar addition; BCF, biochar compound fertilizer.*

The dominant fungal phyla (relative abundance > 5%) across the soil samples were *Ascomycota*, *Mortierellomycota* and *Basidiomycota*, with relative abundances ranging from 30.91 to 49.74%, 29.98 to 50.53% and 7.79 to 15.68%, respectively (Figure 2B). Compared with CK, LB treatment significantly decreased the relative abundance of *Ascomycota* but increased the relative abundance of *Mortierellomycota* in both bulk and rhizosphere soil. At the family level of soil fungi, *Mortierellaceae*, *Nectriaceae*, *Ceratobasidiaceae*, *Bionectriaceae*, and *Trichocomaceae* were abundant (relative abundance > 1%) in all soil samples (Supplementary Figure 3). When compared with CK, the relative abundance of *Mortierellaceae* was significantly increased in LB, HB and BCF for the bulk and increased in CS and LB for the rhizosphere. Moreover, LB treatment significantly increased the Chao1, ACE and Shannon indices of fungi (Table 3).

### Bacterial and Fungal Community Structure and Its Correlation With Soil Properties

**FIGURE 3:**
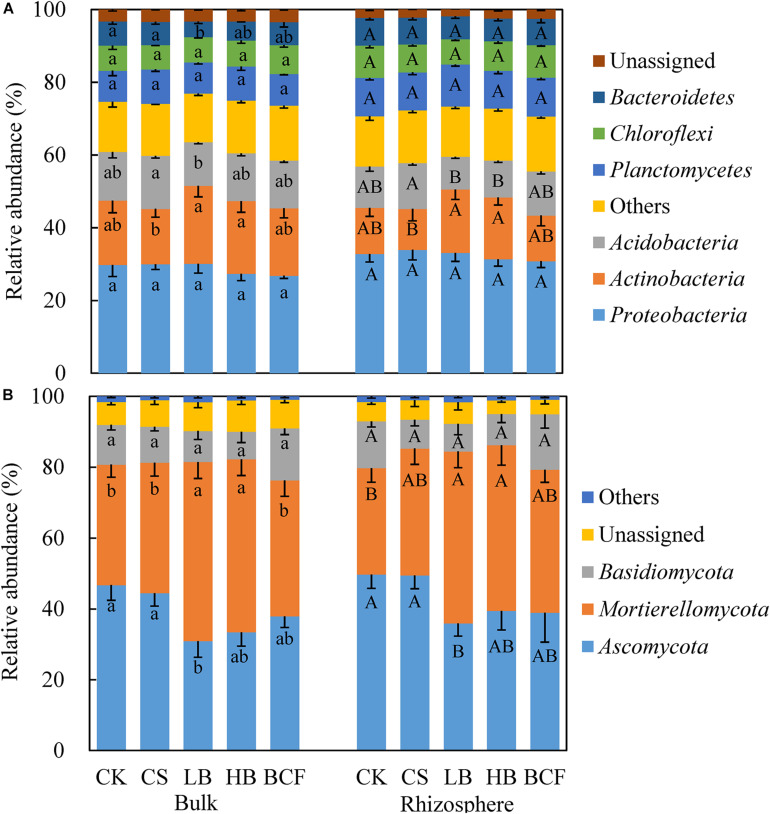
Redundancy analysis (RDA) of the relationship between soil characteristics and community structure of soil bacteria **(A)** and fungi **(B)** in the bulk and rhizosphere soils. The samples were analyzed in triplicate plots.

The effects of soil properties on the microbial communities were analyzed using redundancy analysis (RDA) (Figure 3). These soil variables explained 41.2% of the variety in bacterial communities, with the first two axes explained 28.6% and 12.6% of the total variation, respectively (Figure 3A). For fungal communities, axis1 and axis2 respectively explained 31.8 and 17.5% of the total variation (Figure 3B). Furthermore, soil SOC and TN contents significantly (*p* < 0.05) affected both soil bacterial and fungal community structure. In addition, the effect of soil AK on the fungal community structure was also significant (*p* < 0.05). Similar to RDA results, NMDS showed that bacterial and fungal community compositions separated clearly between biochar and chemical fertilizer regimes (Supplementary Figure 1). For the bacterial community, CK and CS clustered together and separated from biochar treatments (LB, HB, and BCF). For fungal community, CK, CS, and BCF clustered together and separated from LB and HB. This implied that bacterial and fungal community structures were significantly affected by biochar amendment. Furthermore, the community structure of fungi between the bulk soil and rhizosphere were well separated along the NMDS2.

## Discussion

### Effects of Biochar on Soil Properties

Plenty of previous studies have investigated the effects of biochar application through pot experiments or short-term field studies while only a few reported long-term field experiments (Ameloot et al., 2013; Gul et al., 2015; Yao et al., 2017a). Most of these studies reported that biochar amendment improved soil physicochemical properties, such as pH, organic carbon and available nutrients (Atkinson et al., 2010; Lehmann et al., 2011). In a study by Yao et al. (2017a), the application of biochar was observed to significantly increase the soil pH and TN content, especially at a higher application rate (8%). It was similar to the results by Zheng et al. (2016), who reported an increase in pH, SOC and TN after a 4-year field trial with biochar application at a higher rate of 40 t⋅ha^–1^. In this study, we found that the treatment with LB, HB and BCF greatly increased the content of SOC, whereas soil pH showed no significant change. This was due to an initial high pH (8.05) to which biochar amendment brought a slight increase (8.13–8.20) but had no statistical significance (*p* > 0.05). It was also reported that soil pH showed no significant change when biochar was applied in an alkaline field (Luo et al., 2017). As observed in this study and previous studies (Egamberdieva et al., 2016), the labile organic carbon in biochar could be directly utilized by microbes through root exudation. In addition, a significant increase in microbial carbon usage efficiency was observed in biochar-amended soil, thus, promoting the soil SOC retention (Zheng et al., 2016). The observations from current work show that TN and AK content greatly increased with HB treatment. The concentration of AP was also significantly increased with the amendment of biochar and BCF. This suggested that the sorption of N and P by biochar could be a crucial process (Yu et al., 2020). In other studies, these increases were regulated by the available nutrients of biochar and interaction of biochar with fertilizer (Ibrahim et al., 2020). Additionally, Gao et al. (2019) also indicated that biochar when applied alone or in combination with chemical fertilizers could remarkably promote the soil P availability.

### Effects of Biochar on the Abundances of Soil Bacteria and Fungi

Several studies have documented that biochar amendment altered the soil microbial abundance (Chen et al., 2015; Gul et al., 2015). It was also reported that the factors such as soil pH and microbial adhesion, influenced by biochar amendment, indirectly effects the microbial abundance (Lehmann et al., 2011; Cole et al., 2019). Another study also reported an increase in bacterial abundance by biochar addition, especially at a high rate (Yao et al., 2017b). Likewise, Chen et al. (2015) also suggested that bacterial 16S rRNA gene copy numbers increased by 37–60% under biochar amendment at a rate of 40 t⋅ha^–1^, observed with three sites. It should be noted that, various studies showed that soil pH is a dominant factor for the change in bacterial abundance (Yao et al., 2017b; Liu et al., 2019; Zheng et al., 2019; Sun et al., 2020). However, test soil in this study was alkaline, and biochar application resulted no changes in soil pH and bacterial abundance. Similarly, Luo et al. (2017) found that biochar amendment had no significant effect on soil pH in alkaline agricultural soil. In a 2-year field experiment, Soinne et al. (2020) found that soil pH did not differ from the control after the forest residue biochar amendment and there were no differences in bacterial 16S rRNA gene copy numbers between treatments. In this study, the fungal abundance was 8.72–23.56% higher in the rhizosphere than in the bulk soil under all treatments (Figure 1B). This could be due to the divergent properties of rhizosphere, such as root exudations, microbial activity and plant absorption (Philippot et al., 2013). In addition, biochar amendment significantly increased fungal abundance compared with the control. These observations were consistent with the previous studies that showed that biochar application could promote the soil fungal growth (Steinbeiss et al., 2009; Jones et al., 2012; Luo et al., 2017). A similar result was reported by Yao et al. (2017a), who speculated that the possible explanation could be the change in soil pH and nutrient content caused by biochar amendment. In another semiarid farmland trial, a rate of 50 t⋅ha^–1^ (C50) biochar addition remarkably increased the absolute and proportional abundance of fungi, which was ascribed to the increase in SOC (Luo et al., 2017). The current study also reported that biochar amendment greatly increases SOC content and the pairwise correlation analysis showed a significant positive correlation of fungal abundance and SOC (*p* < 0.01).

### Effects of Biochar on the Community Compositions of Soil Bacteria and Fungi

In a 3-year field research, Yao et al. (2017b) revealed that the bacterial diversity was positively correlated with biochar amendment rate when the soil pH was around 6.0. A similar result was reported in a short-term biochar amendment experiment by Hu et al. (2014) where the original soil pH was 3.7. In a number of previous studies, soil pH was frequently reported as a dominant factor effecting bacterial diversity in acidic and neutral soils (Gul et al., 2015; Yao et al., 2017b). However, the soil used in this study was alkaline (pH = 8.19), and biochar amendment resulted no change in soil pH. A similar result was observed by Luo et al. (2017) who found that biochar amended to an alkaline maize field had no impact on soil pH. Moreover, the LB treatment significantly decreased the relative abundance of *Bacteroidetes* compared with the control in the bulk soil, which only accounted for 4.29–7.63% of the total abundance. *Bacteroidetes* species exhibit copiotrophic attributes and were abundant in soil with high C availability (Fierer et al., 2007). Zheng et al. (2016) found that the relative abundances of *Bacteroidetes* decreased by 27–50% in poplar plantation soil compared with the cropland, which may be attributable to the reduction in the organic carbon bioavailability in the poplar plantation soil. In this study, we found that the relative abundance of *Chitinophagaceae* family affiliated with *Bacteroidetes* were significantly decreased in LB treatment compared with the control (Supplementary Figure 2). *Chitinophagaceae* plays an important role in organic carbon decomposition, and decrease in the relative abundance of *Chitinophagaceae* could be attributed to low availability of organic C in biochar amended soils (Zheng et al., 2017a). Though we observed that the concentrations of SOC were increased in the biochar amended treatments, it was likely that the available C rather than the total organic C may play a more important influence on these species, as the dissolved organic C was extremely lower in biochar amended soils compared with the control (Zheng et al., 2017a). Besides, other dominant phyla showed no significant changes with biochar amendment. Thus, the richness and diversity of bacterial community had no significant response to biochar application.

The composition and structure of soil microbial communities is complex and biochar application has been frequently reported to affect the soil microbial community (Atkinson et al., 2010; Xu et al., 2014; Zhang et al., 2017). Studies have demonstrated that a shift in soil properties after biochar amendment, such as soil pH, availability of C, N and other nutrients resulted in the change of microbial community (Lehmann et al., 2011). In this study, NMDS analysis showed that bacterial community in CS treatment clustered together with the CK, which separated from low (LB) and high (HB) biochar treatments, suggesting that biochar application significantly altered the community structure of soil bacteria (Supplementary Figure 1A). In addition, RDA analysis indicated that SOC and TN were two most important factors resulting in a change in bacterial community (Figure 3A). This result was consistent with many previous studies (Luo et al., 2017; Yao et al., 2017b). Likewise, Xu et al. (2014) indicated that TN was the key contributor for shaping the bacterial community structure. Zheng et al. (2016) also revealed that bacterial community in the biochar amended soil was positively correlated with SOC and TN. These studies implied that SOC and TN could alter the bacterial community compositions at the phylum level. Compared to CK, the relative abundance of *Actinobacteria* in the rhizosphere was increased by 37.35 and 33.69% under LB and HB treatments, respectively (Figure 2). It is known that *Actinobacteria* is a group of growing readily on the carbon-rich material and related to decompose the organic materials or substrates in biochar-amended soil (O’Neill et al., 2009; Chen et al., 2015). The increase of *Actinobacteria* in this study could be attributed to the biochar addition which not only adds nutrients like carbon source into the soil, but also stimulates the growth and multiplication of bacteria (Anderson et al., 2011; Lehmann et al., 2011).

In contrast, the LB treatment greatly increased the OTUs and fungal alpha diversity indices, including Chao1, ACE, and Shannon, regardless of the bulk soil or rhizosphere. This suggested that the response of different microbial groups to biochar amendment was varied, which was also consistent with previous research (Yao et al., 2017a, b). In a 133 days incubation experiment, hydrochar addition distinctly increased fungal diversity compared to the control, especially at a rate of 30 t⋅ha^–1^ (Sun et al., 2020). Moreover, Zheng et al. (2016) and Cheng et al. (2019) revealed that the porous structure of biochar could provide a more suitable refuge for fungi to grow and protect them from predators. Biochar contains lower concentrations of N, P, K and other available nutrients compared to other organic matter, but could stabilize these elements in substrate ascribed to its porous structure and strong adsorption (Liu et al., 2016). On the other hand, it was suggested that fungi might have more readily available nutrients by absorbing to biochar (Meng et al., 2019).

Similar to bacteria, fungal community structure was significantly affected by biochar application (Supplementary Figure 1A). The fungal community structure was found to be distinct between the bulk soil and rhizosphere. Rhizosphere microbes could be affected by root exudations and plant absorption (Philippot et al., 2013; Wang et al., 2018). On the other hand, fungi are more sensitive to rhizosphere than bacteria (Hu et al., 2014; Yao et al., 2017a). Previous short-term experiments have shown that biochar amendment altered the fungal relative abundances at the phylum level (Chen et al., 2013; Hu et al., 2014). In this study, the dominant fungal phyla across all soil samples were *Ascomycota*, *Mortierellomycota*, and *Basidiomycota*. The LB treatment significantly decreased the relative abundance of *Ascomycota* phyla but increased the relative abundance of *Mortierellaceae* family when compared to the control. Moreover, it was also found that *Ascomycota* significantly decreased by 11% after 40 t⋅ha^–1^ biochar amendment and this may be attributed to co-variations with soil pH, SOC, and C/N (Zheng et al., 2016). Their study also discovered that *Mortierella* was greatly enhanced by biochar amendment especially at a high application rate and was positively correlated with SOC. It was consistent with the results from our study where RDA analysis indicated that the three most important contributors to fungal community variations were SOC, AK, and TN (Figure 3B). Changes in the availability of soil nutrients caused by biochar addition could alter the soil enzyme activities (Marschner, 2003). These reasons ultimately resulted in the changes in the abundance and community composition of soil fungi.

## Conclusion

Our study demonstrated that the amendment of biochar and BCF brought significant changes in soil properties and community structure of both bacteria and fungi after 3 years amendment. The abundance and diversity of both bacteria and fungi was higher in the rhizosphere than that of the bulk soil. Fungal community was more sensitive to biochar application compared with soil bacteria. After 3 years, biochar amendment increased soil fungal abundance and diversity, and shifted fungal community structure. RDA analysis indicated that changes in bacterial and fungal communities were significantly affected by SOC and TN contents, suggesting that biochar altered microbial communities in a direct or indirect way. However, as a soil improving material, further studies are needed to focus on the long-term impacts of biochar on the microbial community with specific soil functions.

## Data Availability Statement

The datasets presented in this study can be found in online repositories. The names of the repository/repositories and accession number(s) can be found below: NCBI (accession: PRJNA702237).

## Author Contributions

YL, XL, and LJ designed the research. WG, KG, and ZG performed the data analysis. WG wrote the manuscript. WG, LJ, CL, and GW revised and commented on the draft. All authors read and approved the final manuscript.

## Conflict of Interest

The authors declare that the research was conducted in the absence of any commercial or financial relationships that could be construed as a potential conflict of interest.

## Abbreviations

qPCR: quantitative real-time PCR
OTU: operational taxonomic unit
NMDS: non-metric multidimensional scaling
RDA: redundancy analysis
ITS: internal transcribed spacer.
