# Supplementary material for: Different Responses of Soil Bacterial and Fungal Communities to 3 Years of Biochar Amendment in an Alkaline Soybean Soil
Source: Front Microbiol. 2021 May 26;12:630418. doi: 10.3389/fmicb.2021.630418 (PMC8187762; doi:10.3389/fmicb.2021.630418)
Supplement: Supplementary file 1 [file Data_Sheet_1.docx]

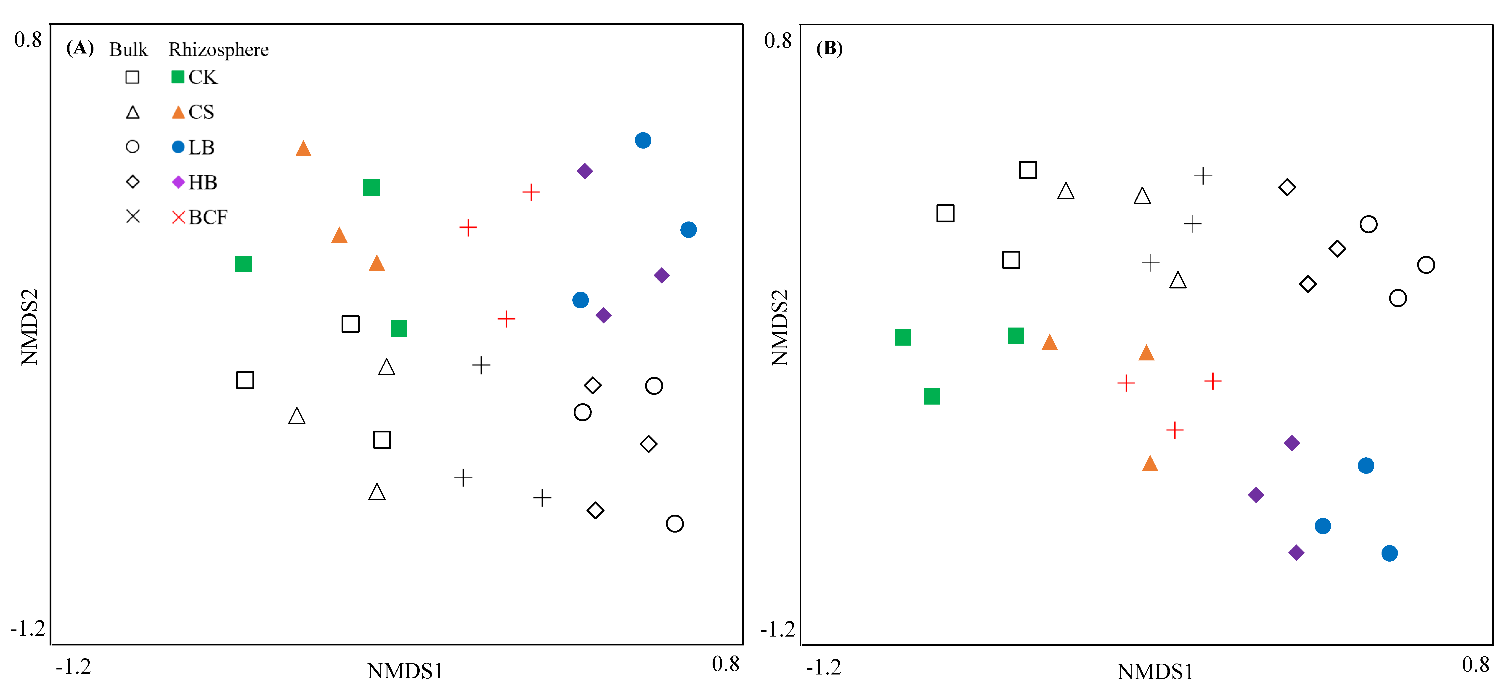
**Supplementary Information:**

**Fig. S1** Non-metric multidimensional scaling (NMDS) analysis of soil bacteria (A) and fungal (B) community structure in the bulk and rhizosphere soil of soybean.


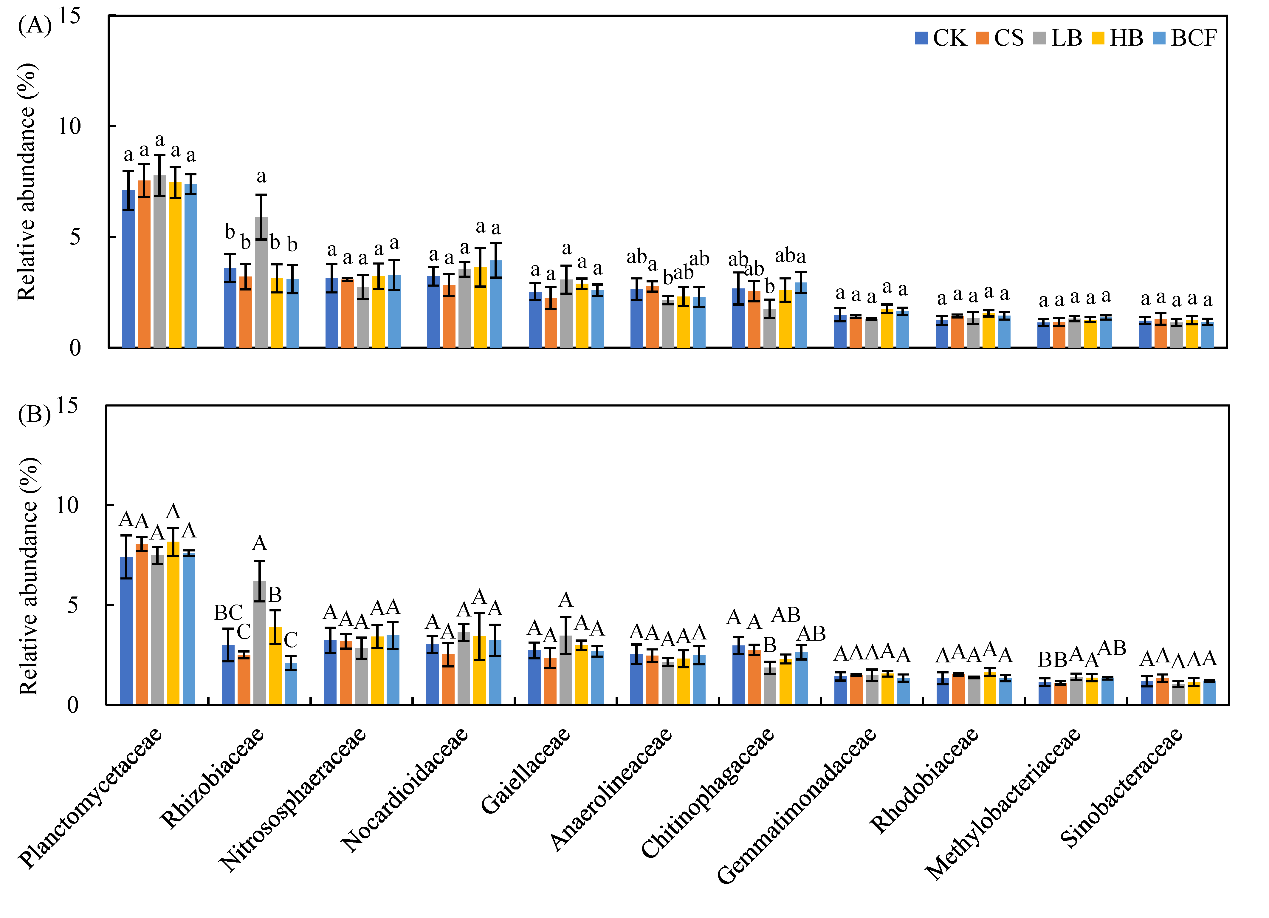


**Fig. S2** Relative abundance (%) of the dominant family of bacteria in the bulk (A) and rhizosphere (B) soils of soybean. Different letters above the columns denote significant differences among the treatments in the bulk or rhizosphere soil at *p* < 0.05, lowercase letters for the bulk soil and uppercase letters for the rhizosphere. CK: control; CS: wheat straw addition; LB: low biochar addition; HB: high biochar addition; BCF: biochar compound fertilizer.


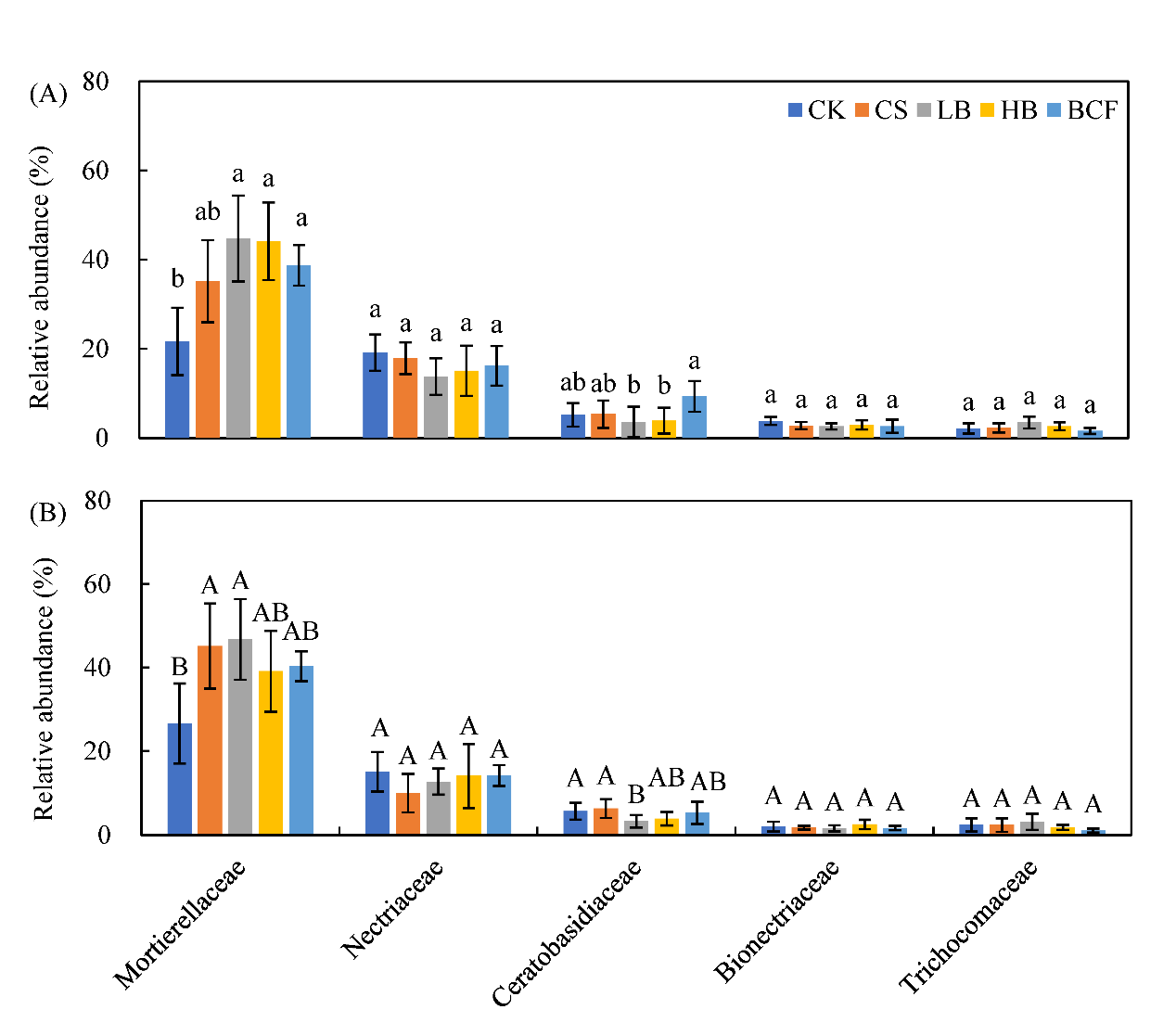


**Fig. S3** Relative abundance (%) of the dominant family of fungi in the bulk (A) and rhizosphere (B) soils of soybean. Different letters above the columns denote significant differences among the treatments in the bulk or rhizosphere soil at *p* < 0.05, lowercase letters for the bulk soil and uppercase letters for the rhizosphere. CK: control; CS: wheat straw addition; LB: low biochar addition; HB: high biochar addition; BCF: biochar compound fertilizer.
